# Molecular Assessment of Resistance to Clarithromycin in *Helicobacter pylori* Strains Isolated from Patients with Dyspepsia by Fluorescent In Situ Hybridization in the Center of Iran

**DOI:** 10.1155/2020/2304173

**Published:** 2020-03-27

**Authors:** Jina Vazirzadeh, Jamal Falahi, Sharareh Moghim, Tahmineh Narimani, Rahmatollah Rafiei, Vajihe Karbasizadeh

**Affiliations:** ^1^Department of Microbiology, School of Medicine, Isfahan University of Medical Sciences, Isfahan, Iran; ^2^Health Clinical Sciences Research Center, Zahedan Branch, Islamic Azad University, Zahedan, Iran; ^3^Islamic Azad University, Najafabad Branch, Iran

## Abstract

**Background and Aims:**

*Helicobacter pylori* is a common infectious bacterium mostly found in gastroduodenal diseases. The increased prevalence of clarithromycin-resistant *H. pylori* strains is a major challenge in the successful treatment of infections caused by this organism. The present study is aimed at detecting the clarithromycin resistance pattern of *H. pylori* strains isolated from gastric biopsies and evaluating point mutations of the 23S rRNA gene. *Patients and methods*. In the present descriptive cross-sectional study, 165 patients with gastrointestinal disorders, who were referred to the Endoscopy Center of Dr. Shariati Hospital of Isfahan, Iran, were enrolled from April to July 2018. *H. pylori* infection was diagnosed by culture, and susceptibility of the isolates to clarithromycin was assessed by the E-test. Minimum inhibitory concentration (MIC) values were obtained based on EUCAST recommendations. Also, fluorescence in situ hybridization (FISH) was used to determine point mutations associated with clarithromycin resistance.

**Results:**

By using culturing, *H. pylori* was isolated from 50.3% (83/165) gastric biopsy specimens. The overall frequency of resistance to clarithromycin was 25.3% (21/83) by the E-test. In the resistance genotypic analysis, 19 isolates had mutations. The prevalence of A2143G and A2144G mutations was 68.4% (13/19) and 31.5% (6/19), respectively. A2143C mutation was not tracked in any isolate. Two isolates with MIC > 0.5 *μ*g/mL had no mutations that could be related to other mechanisms of resistance.

**Conclusion:**

As presented in the study, the high prevalence of clarithromycin-resistant *H. pylori* due to point mutations of the 23S rRNA gene indicates the necessity of revising the standard treatment regimen based on antibiotic susceptibility pattern of each region.

## 1. Introduction

*Helicobacter pylori* (*H. pylori*) is a common pathogenic bacterium worldwide including Iran. It is claimed to cause persistent gastrointestinal infection by colonization in the human gastric mucosa [1, 2]. In the case of untreated and prolonged infection, *H. pylori* can cause peptic ulcer disease (PUD) and chronic gastritis (CG). Furthermore, neoplastic processes such as mucosa-associated lymphoid tissue (MALT) and gastric cancer (GC) are associated with this microorganism [3, 4]. The International Agency for Research on Cancer classified *H. pylori* as class I carcinogen in 1994 [5]. Following the successful treatment, the risk of GC decreases in patients who lack the premalignant lesions. According to the consensus of most guidelines, eradication therapy is essential for symptomatic patients [6].

Standard triple therapy consists of a proton pump inhibitor (PPI) in combination with two antibiotics: clarithromycin, metronidazole, or amoxicillin is considered to cure *H. pylori* infection. Based on the Maastricht V consensus reports, the above treatment is appropriate in countries with less than 15% resistance to clarithromycin. There are numerous, important factors (human and bacterial) in reducing the effect of *H. pylori* treatment, of which the antibiotic resistance seems to be the most significant [7, 8].

Clarithromycin is a key antibiotic in the combination therapy, and resistance of it has been reported as a major cause of failure in the eradication therapy. In Iran, the resistance to this antibiotic is 22.4% on average. However, the eradication is lower in Iran than in developed countries [9, 10]. Clarithromycin binds to the 50S ribosomal subunit in the 23S rRNA gene and inhibits protein synthesis. Antibiotic resistance mechanisms are mainly related to point mutations in the region of domain V of 23S rRNA. The most common mutations have been identified in the A2142G and A2143G positions. However, several other mutations have been detected in A2144G, A2143C, and T2182C positions. Given the heterogeneous distribution of antibiotic resistance across different geographical areas, determining regional patterns of antibiotic resistance can help the physicians choose a valuable and specific treatment [11, 12].

Phenotypic assays of determining antibiotic susceptibility including gradient diffusion susceptibility testing (E-test) and the agar dilution method are time-consuming, expensive, and not routine [13]. Therefore, molecular-based techniques such as PCR-restriction fragment length polymorphism (RFLP), real-time PCR, and fluorescence in situ hybridization (FISH) are appropriate alternatives to the phenotypic methods and can also detect point mutations associated with the antibiotic resistance. FISH is a new technology based on fluorescently labeled DNA probes that hybridize with specific rRNA sequences of microorganisms and can identify *H. pylori* and resistance to clarithromycin in formalin-fixed paraffin-embedded tissue sections or cultured *H. pylori* colonies [13, 14].

The main objective of the present study was to determine the clarithromycin resistance pattern in *H. pylori* strains isolated from the gastric biopsy of patients with dyspepsia and evaluate point mutations in the 23S rRNA gene in Isfahan, Iran.

## 2. Methods

### 2.1. Patients

This cross-sectional descriptive study was conducted in Dr. Shariati Hospital of Isfahan, Iran, as a participating center in the Global Antimicrobial Resistance Surveillance System (GLASS) Project of the World Health Organization (WHO). A total of 165 patients who were referred to the center at the time with clinical symptoms of gastrointestinal disorders, selected by inclusion and exclusion criteria which are explained in the following sentences, were recruited into the study and admitted to the outpatient Gastroenterology Clinic and Endoscopy Unit.

The inclusion criteria were upper abdominal pain, dysphagia, nausea, vomiting, dyspepsia, and gastroesophageal reflux.

The exclusion criteria were treatment with antibiotics or taking any proton pump inhibitor (PPIs), H2-receptor blocker, and nonsteroidal anti-inflammatory drugs (NSAIDs) within the 2 weeks prior to endoscopy.

These patients underwent gastric endoscopy from April 2018 to July 2018. The study was approved by the Research Ethics Committee of the Isfahan University of Medical Sciences (no. IR.Mui.rec.1396.3.878), Isfahan, Iran. Written informed consent was obtained from all the gastroduodenal patients.

### 2.2. Endoscopy and Gastric Biopsy Sampling

Gastric biopsy specimens were obtained from the antrum and corpus of the stomach during each endoscopic procedure by the use of sterile biopsy forceps. One set was placed in sterile Eppendorf tubes, containing 1 mL sterile physiological solution (0.9%Nacl), and immediately transported to the microbiology laboratory for culturing *H. pylori* in a microaerophilic environment. A second set was fixed and transported in 10% buffered formalin for histopathological examination and FISH. A skilled pathologist evaluated the biopsy samples.

### 2.3. *H. pylori* Culture

Biopsy specimens were sent to the Clinical Microbiology Lab within half an hour of sampling in sterile tubes. Then, biopsies were homogenized in saline and inoculated on selective medium Columbia agar (Gibco, USA) supplemented with 5% defibrinated sheep blood, 10% fetal calf serum (FCS), and campylobacter selective supplement (Merck, Germany). The plates were incubated for 5-7 days at 37°C under microaerophilic conditions (Anoxomat; MART Microbiology BV, Drachten, the Netherlands). *H. pylori* was identified based on colony morphology (small and translucent colonies) and typical appearance on Gram stain as a gull wing-shape bacteria and also by positive reactions for oxidase, catalase, and strong urease activity. After subculturing on Columbia agar, all of them were stored in brain heart infusion broth with 30% glycerol and 7% FCS at −70°C [15, 16].

### 2.4. Antibiotic Susceptibility Testing

The E-test was applied for *H. pylori* strains, using strips of clarithromycin, amoxicillin, and metronidazole (Liofilchem, Italy) on Mueller-Hinton agar (MHA; Merck, Germany) which was enriched with 7% defibrinated sheep blood. Bacterial colonies obtained during 72-hour culture were suspended in sterile physiological saline (10^8^ cells CFU/mL; turbidity, 3 McFarland) and streaked onto MHA with a suspension-immersed cotton swab. The strips were placed on the medium after drying the medium surface. The plates were then incubated under microaerophilic conditions for 72 hours at 37°C. The value of minimum inhibitory concentration (MIC) was measured according to the recommendations of the European Committee on Antimicrobial Susceptibility Testing (http://www.eucast.org/clinicalbreakpoints/). The breakpoint clarithromycin resistance was >0. 5 *μ*g/mL [17]. MIC values were classified into low (MIC from >0.5 to ≤8 *μ*g/mL) and high (MIC from >8 to 256 *μ*g/mL) groups [18].

The breakpoint amoxicillin resistance > 0.125 *μ*g/mL and metronidazole resistance > 8 *μ*g/mL were considered. ATCC 43504 was used as the reference strain for the quality control of antibiotic susceptibility testing.

### 2.5. Detection of Point Mutations in the *H. pylori* 23S rRNA Gene by FISH

#### 2.5.1. Oligonucleotide Probes

Oligonucleotide probes were synthesized (Metabion, Munich, Germany) and 5′ labeled with fluorescein isothiocyanate (Hpy-1; green signal) or the fluorochromes Cy3 (ClaR1, ClaR2, ClaR3, and ClaWT; red signal). Probe ClaWT was used as an internal control to identify clarithromycin-sensitive *H. pylori* isolates which had not been detected by the mixture of probes ClaR1, ClaR2, and ClaR3 [19]. The probes, their respective sequences, and locations within the rRNA operons are summarized in Table 1.

#### 2.5.2. Preparation of Gastric Biopsy Specimens

Within several hours after endoscopy, fixed biopsy specimens were embedded in paraffin, cut into 4 *μ*m thick sections, and placed on glass slides. The specimens were incubated at 55°C overnight. To deparaffinize the tissue sections, the slides were subsequently immersed in hexane and absolute ethanol (Merck, Germany) twice, each time for 30 minutes. After air-drying of the slides, they were then ready for checking by FISH [12].

#### 2.5.3. FISH

At first, each slide of the tissue sections was overlaid with 50 *μ*L of hybridization buffer (0.9 M NaCl, 0.02 mM Tris-HCl (pH 8.0), 0.01% sodium dodecyl sulfate, and 30% formamide) and an oligonucleotide mixture (5 ng/mL) consisting of the probes Hpy-1, ClaR1, ClaR2, and ClaR3; ClaR1, ClaR2, and ClaR3; or Hpy-1. Hybridization was carried out at 46°C for 90 min in a humid chamber, and stringent washing was done at 48°C in a buffer containing 0.112 M NaCl, 20 mM Tris-HCl (pH 8.0), and 0.01% sodium dodecyl sulfate. The slides were then stained with 1 *μ*g/mL DAPI (4′,6-diamidine-2′-phenylindole hydrochloride) for 5 minutes. DAPI nonspecifically stains the DNA of any cell, including bacteria, blue. Finally, the slides were washed with PBS, left to air-dry, covered with fluorescent mounting medium (DAKO, Denmark), and examined with an epifluorescence microscope (BX61 Olympus, Hamburg, Germany) equipped with different filters. Microscopy was carried out by two blinded independent investigators. Antibiotic-resistant strains were visible in yellow (a mixture of green and red).

Determination of the common point mutations (A2143G, A2144G, and A2143C) causing resistance to clarithromycin in *H. pylori* strains was performed in clarithromycin-resistant specimens that had shown a positive result with the mixture of the probes. Samples were examined separately with each probe (ClaR1, ClaR2, and ClaR3), to differentiate between single-point mutations.

At the same time as the mutation was determined in the clarithromycin-resistant strains, the positive and negative control slides, respectively, were obtained from the mutant (resistant) and wild (susceptible) strains, prepared from Ahvaz Jundishapur University of Medical Sciences. FISH was performed according to the protocol mentioned above. Some mutant strains were randomly reexamined [20].

### 2.6. Statistical Analysis

The Student *t*-test was used for comparison between means. Nominal data were analyzed using chi-square test, and *P* values < 0.05 were considered statistically significant. The agreement between different diagnostic tests was evaluated by calculating Cohen's kappa confidence. All analyses were performed using SPSS Statistics version 20 (SPSS, Chicago, USA).

## 3. Results

### 3.1. *H. pylori* Infection Rate

**Figure 1:**
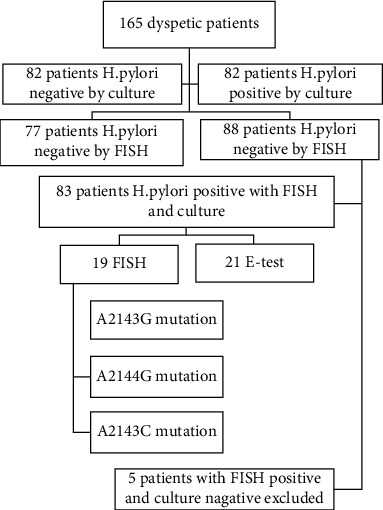
Flowchart of patients.

A total of 165 patients, consisting of 84 males and 81 females, were enrolled in this cross-sectional study. The mean age was 50.3 ± 15.5 years old with the age ranging from 15 to 83 years. The overall prevalence of *H. pylori* based on the culture and FISH methods was 50.3% (83/165). In other 5 biopsy specimens, *H. pylori* was only detected by the FISH, but not by culture. Therefore, 83 patients were analyzed (Figure 1). In a pathological finding, there were 40 (48%) chronic gastritis, 34 (41%) chronic active gastritis, and 9 (11%) erosive gastritis patients.

### 3.2. Antibiotic Susceptibility Profile

Due to the suggested breakpoints of the EUCAST, clarithromycin-resistant *H. pylori* isolates were diagnosed in 25.3% (21/83) patients by the E-test. Based on the values of the MIC, 47.61% (10/21) of isolates showed high-level resistance and 52.38% (11/21) had low-level resistance. The frequency of clarithromycin resistance was 25% (10/40) in chronic gastritis, 26.5% (9/34) in chronic active gastritis, and 22.2% (2/9) in erosive gastritis. We found no significant association between the presence of resistant strains and different types of dyspeptic disorders (*P* = 0.96). The clarithromycin resistance was different between females (32.5%) and males (18.6%), but the difference did not reach statistical significance (*P* = 0.14). In relation to age, no statistically significant difference was identified between the mean age of the patients with resistance strains (51.48 ± 15.41) and of the remaining infected patients (49.63 ± 14.73) (*P* = 0.62).

Frequencies of resistance rates of 83 isolates to selected antibiotics were 66.2% (55/83) and 18.07% (15/83), for metronidazole and amoxicillin, respectively.

### 3.3. Mutations of the 23S rRNA Gene

**Figure 2:**
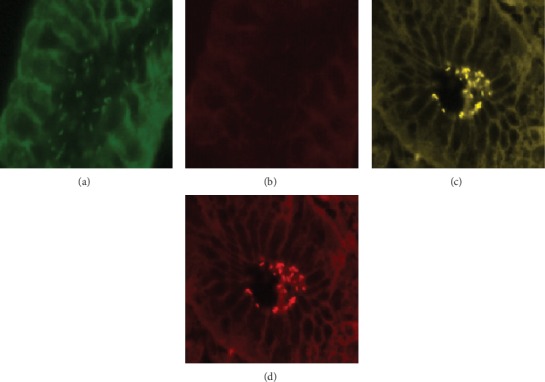
Specific detection of clarithromycin-sensitive and clarithromycin-resistant *H. pylori* isolates in gastric biopsy specimens by FISH. (a) Identification of bacteria in green, due to hybridization with probe Hpy-1–FITC. (b) Visualization of the same microscopic field in red cannel, indicating strain is clarithromycin-sensitive *H. pylori*. (c) Demonstration of clarithromycin-resistant *H. pylori* in the biopsy specimen of a patient by simultaneous application of probe Hpy-1–FITC and the mixture of probes ClaR1-Cy3, ClaR2-Cy3, and ClaR3-Cy3; bacteria are visible in yellow (mixed color of green and red). (d) Visualization of the same microscopic field in red cannel, due to hybridization with probe ClaR1-Cy3, ClaR2-Cy3, or ClaR3-Cy3.

The analysis of mutations in the 23S rRNA gene revealed mutation in 22.9% (19/83) isolates. Among these strains, 68.4% (13/19) had mutation at position A2143G. In 31.5% (6/19) of strains, mutation at position A2144G was detected. Furthermore, A2143C was found in none of the strains. No point mutation in the 23S rRNA gene was detected in 62 of the clarithromycin-susceptible isolates (Figure 2). In none of the gastric biopsy specimens, two strains of *Helicobacter pylori* (one clarithromycin-sensitive strain (hybridization with probe Hpy-1) and one clarithromycin-resistant strain (hybridization with the probe mixture of Hpy-1–ClaR1–ClaR2–ClaR3)) were identified. Also, two or more different mutations were not observed in one strain at the same time, and there was only one type of mutation in each of the 19 resistant strains.

In two isolates, which had MIC > 0.5 based on the E-test method, the examined mutations were not detected by the FISH and were named as “undetermined.” The agreement between the results of E-test and FISH by Cohen's kappa coefficient was found substantial (kappa = 0.93). According to the cutoff point of the study, 69.2% of strains had MIC > 8 with A2143G mutations, while 83% of strains had MIC < 8 with A2144G mutations (Table 2). Also, analysis association between mutation type with a pathologic finding of patients and the gender of the patients showed that there were no statistically significant differences (*P* = 0.058 and *P* = 0.63, respectively).

## 4. Discussion

The prevalence of *H. pylori* infection in the adult population of Iran reaches up to 80% [21]. Increasing antibiotic resistance in recent years has reduced the efficacy of treatment regimens. Clarithromycin resistance is the main cause in the failure of *H. pylori* infection eradication.

The first relevant finding of the present study is that the resistance rate of clarithromycin was 25.3% in *H. pylori* strains. We observed a higher percentage of clarithromycin resistance compared to previously published studies [22, 23]. This finding indicates that the current *H. pylori* clarithromycin resistance rate in this region has reached a high level. Studies from the USA, Asia, and Europe reported the rate of resistance in 29.3%, 58.8%, and 42.35% of the strains, respectively [24, 25].

Unfortunately, different regions worldwide including Iran face increasing drug resistance annually. Antibiotic resistance of a drug is a reflection of its pattern of use in any geographical region. Macrolides in Iran are widely prescribed by physicians for the treatment of respiratory infections. It led to the induction of cross-reactivity to clarithromycin and increased resistance to this drug. Therefore, alternative medication regimens (e.g., quadruple therapy or modified triple regimens) should be considered for effective eradication therapy in this area. Clarithromycin resistance is associated with point mutations of the V domain of 23S rRNA in the 50S subunit that decreases the affinity of drug binding to the ribosome [4, 6]. In our study, A2143G was the most common type of mutation in strains. This finding verified previous studies by other researchers [7, 26]. Furthermore, 9.5% of clarithromycin-resistant *H. pylori* strains lacked the studied mutations. These isolates were considered undetermined clarithromycin-resistant strains. The resistance of these isolates may be related to the existence of mechanisms unrelated to the 23S rRNA gene sequence, such as expression of the RND efflux pumps (resistance nodulation-cell division) and other mutations (e.g., A2142G and T2182C) which were not investigated in our study [27].

Another finding of this research indicated that 69.2% of strains with A2143G mutation had the MIC of 8 to 256 *μ*g/mL; however, 83.3% of strains with A2144G mutation had a MIC of 0.5 to 8 *μ*g/mL. Hanafiah et al. reported that more than 40% of strains with A2143G mutation had a MIC of 64 to >256 *μ*g/mL [7]. The association of MIC values and point mutations in 23S rRNA gene was first determined by Versalovic et al. who suggested the MIC value of 64 *μ*g/mL as a cutoff point between high and low levels of antibiotic resistance, and they had high-level resistance in 84% of strains with the A2143G mutations and low-level resistance in 58% of A2144G mutations [28]. A2143G mutation not only led to high MIC value but also might have increased the risk of failure of the *H. pylori* treatment [29]. However, Kim et al. did not obtain any correlation between the mutation type and the clarithromycin MICs [30]. Quek et al. revealed that A2143G and T2182C genotypes were detected not only in resistant isolates but also in clarithromycin-susceptible strains [31]. Chen et al. isolated two clarithromycin-susceptible *H. pylori* strain with mutation A2143G of the 23S rRNA gene [15].

The abovementioned studies suggest that multiple and synergic genetic mechanisms should be taken into account to judge the phenotypic resistance of an isolate, and the antibiotic resistance should not be considered solely dependent on the presence of a specific mutation. Therefore, it is not yet clear whether the determination of types of point mutations can be a strong prognostic factor for clarithromycin resistance and MIC values. We believe that further research is required to clarify the role of point mutations in the molecular mechanism of clarithromycin resistance on a wider population to make an accurate judgment.

In some reports, risk factors of resistance to clarithromycin have been investigated. In this study, although the relative frequency of clarithromycin resistance was higher in females than males, the difference was not significant. This is consistent with the findings of others [12, 32]. The consumption of new macrolides, which are commonly prescribed in the elderly, can lead to more mutations. In the present study, there was no significant relationship between resistance and aging. Zaki et al. showed a significant correlation between resistance status and age [25]. Hence, further research on the relationship between age and antibiotic resistance with larger sample sizes is recommended. We also considered the correlation between pathologic findings and resistance to clarithromycin. A high relative frequency of resistance was seen in CAG patients; however, there was no significant relationship between various diseases and clarithromycin resistance status. These findings are in agreement with Eghbali et al. [32].

In the present research, samples were collected from patients referred to a large teaching hospital in Isfahan. Therefore, the population of the participants may not be the representative of all individuals and the prevalence of antibiotic resistance in this geographical region. Furthermore, the present study did not investigate the genotypic resistance to other antibiotics (e.g., metronidazole and amoxicillin) that were widely used in empirical therapy.

## 5. Conclusion

The prevalence of resistance to clarithromycin of clinical *H. pylori* strains is increasing in this region. Thus, understanding the antibiotic susceptibility pattern helps modify treatment strategies. The implementation of reliable and rapid molecular methods can affect the accurate diagnosis and management of *H. pylori* infection treatment and reduce the incidence of gastric cancer. Furthermore, performing the antibiotic susceptibility test by physicians before prescribing first-line antibiotics can reduce the distribution of secondary resistance, especially to clarithromycin.

## Figures and Tables

**Table 1:** Sequences of the different oligonucleotide probes for detection of *H. pylori* and clarithromycin resistance.

Name	Sequence (5′-3′)	Target site	Specificity
Hpy-1	CACACCTGACTGACTATCCCG	16S rRNA	*H. pylori*
ClaR1	CGGGGTCTTCCCGTCTT	23S rRNA	A2143G (ClaR)
ClaR2	CGGGGTCTCTCCGTCTT	23S rRNA	A2144G (ClaR)
ClaR3	CGGGGTCTTGCCGTCTT	23S rRNA	A2143C (ClaR)
ClaWT	CGGGGTCTTTCCGTCTT	23S rRNA	Wild type (ClaS)

**Table 2:** Association between clarithromycin MICs and point mutations in 23S rRNA gene *H. pylori*.

Type of mutation	Number (%) of isolates		Total
	Low resistance no. (%)	High resistance no. (%)	
A2143G	4 (36.4%)	9 (90%)	13 (62%)
A2144G	5 (45.4%)	1 (10%)	6 (28.6%)
Undetermined	2 (18.2%)		2 (9.5%)

MICs: minimum inhibitory concentrations.

## Data Availability

The data used to support the findings of this study are included in the article.
